# Case Report: Hepatic infantile hemangioma malignantly transformed into hemangiosarcoma

**DOI:** 10.3389/fonc.2025.1614698

**Published:** 2025-08-20

**Authors:** Xiafei Gu, Qing Tao, Zijian Lu, Jianping Liu, Zhang Zhang, Changli Lu

**Affiliations:** Department of Pathology, West China Hospital, Sichuan University, Chengdu, China

**Keywords:** liver, infantile hemangioma, hemangiosarcoma, malignant transformation, tumor

## Abstract

In this study, we retrospectively analyzed the clinicopathological features of a case of hepatic infantile hemangioma (HIH) that malignantly transformed into hemangiosarcoma. HIH, a congenital disease, is the most common benign tumor of the liver in children, and its malignant transformation into hepatic angiosarcoma (HAS) is rare. HIH expresses markers of vascular origin and specifically expresses glucose transporter protein isoform 1. When the malignant transformation of HAS occurs, the vascular lumens anastomose with each other and form a sieve mesh, with pseudo-papillae and solid areas, heterogeneous proliferation of tumor cells, spindle/or epithelioid morphology, heterogeneous distribution of chromatin, enlarged nuclei, and pathologic karyotypes, with strong diffuse positivity of P53 and P16, increased Ki-67 proliferative activity, suggest malignant transformation of HAS. Imaging is the preferred examination method for HIH; however, the presence or absence of characteristic changes in HAS is unclear. There is no uniform treatment guideline for this type of tumor, and a reasonable individualized treatment plan should be formulated according to the specific conditions of the children. There is a lack of case reports of the malignant transformation of HIH into HAS. It is unclear whether HIH treatment should be still extended, and the prognosis is also unclear; therefore, more case reports are needed to accumulate experience.

## 1 Introduction

Currently, although there are more than 340 reports related to HIH, there exist only 9 reports about malignant transformation into HAS, and only 23 reports related to the pathology of HIH, with even fewer reports related to the pathology of HIH with malignant transformation into HAS. HIH, once known as infantile hemangioendothelioma, is a congenital disease and is the most common benign tumor of the liver in children, accounting for 2–3% of all childhood tumors (1), and is most common in infants aged <1 year. Approximately 90% of cases present with signs and symptoms within the first 6 months of life, and there are more female children among those affected, compared with male children. The etiology of the disease is unknown, with only a few reports of a possible association with elevated environmental arsenic exposure and follicle-stimulating hormone secretion (2, 3). Clinical manifestations are varied and not clearly specific, depending mainly on the size and location of the tumor and its complications. The typical clinical trial is hepatomegaly, congestive heart failure, and anemia (4). A common symptom is an abdominal mass, which can be combined with thrombocytopenia sign (Kasabach–Merrit syndrome) in 40% of the patients (5), and approximately 10% of cases are accompanied by hemangiomas of the skin or other areas (which may present in combination with vascular malformations of the skin, brain, gastrointestinal tract, and other organs), and may also present with fear of food, vomiting, slow growth, jaundice, and fever. Some children may have elevated serum alpha-fetoprotein levels (6, 7). Some patients are at high risk of complications such as abdominal compartment syndrome, HF (8), and severe hypothyroidism (9). These serious complications contribute to tumor-related mortality; once the tumor is resected, these adverse risk factors are corrected. The greater the number of complications, the worse the prognosis (10).

## 2 Case description

A 2-year-old child with abdominal pain, fever, and multiple occupations in the liver was admitted to our hospital in June 2023.The patient presented with a height of 95 cm and a weight of 14 kg, along with hypothyroidism-triiodothyronine (T3) 0.51 nmol/L, free triiodothyronine (FT3)2.37 pmol/L, thyroxine (T4)56.60 nmol/L, free thyroxine (FT4)11.90 pmol/L.Laboratory findings indicated a hemoglobin level of 102 g/L. The tumor marker CA125 was elevated (35.60 U/mL), whereas alpha - fetoprotein (AFP), carcinoembryonic antigen (CEA), and carbohydrate antigen 19 - 9 (CA19 - 9) were all within normal limits. Abnormal prothrombin was normal, and serological tests for hepatitis B, syphilis, hepatitis C, and HIV yielded negative results. Liver function tests showed an elevated level of alkaline phosphatase (ALP; 139 IU/L), while alanine aminotransferase (ALT), aspartate aminotransferase (AST), and gamma - glutamyl transferase (GGT) were all within normal ranges.

**Figure 1:**
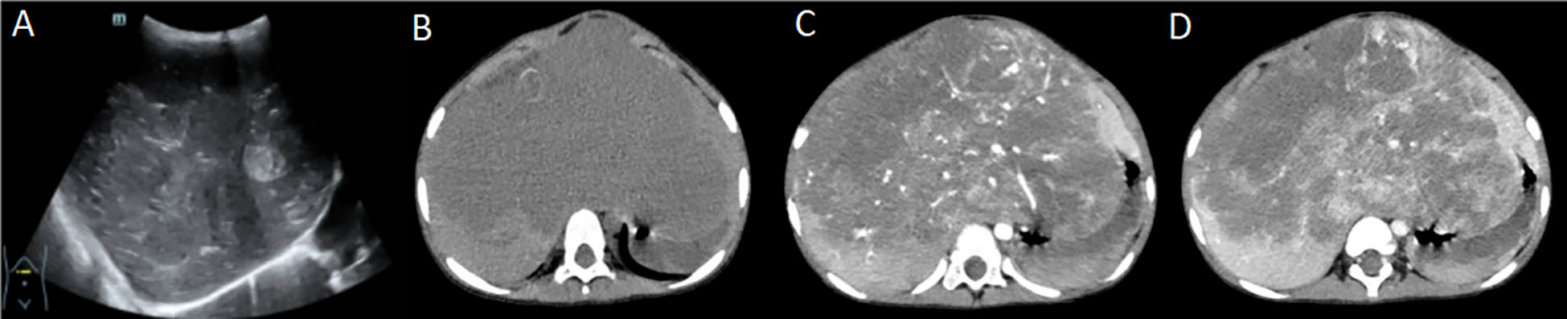
**(A)** Liver ultrasound; **(B)** Liver computed tomography (CT) - plain image; **(C)** Liver CT enhancement scan - arterial phase; **(D)** Liver CT enhancement scan - portal venous phase (part of the portal venous system is not well displayed due to tumor encapsulation).

In August 2023, he was admitted to our hospital with a large occupational mass in the epigastric region and hypothyroidism. Color ultrasound examination of the liver showed abnormal liver morphology, slightly uneven parenchymal echogenicity. It also revealed multiple slightly echogenic, weakly echogenic, and isoechoic masses in the liver, larger than 13.1 × 9.3 × 8.1 cm, with unclear borders, irregular morphology, partly fused with each other, with slightly rich blood flow signals in some parts. The left branch of the portal vein was encircled by the intrahepatic masses, which was indicative of a neoplastic lesion but did not exclude hepatoblastoma (**Figure 1A**). A computed tomography (CT) scan of the liver showed a huge soft tissue density mass in the liver, with uneven density and unclear boundaries (**Figure 1B**). An enhanced scan showed obvious uneven enhancement in the arterial phase (**Figure 1C**), further enhancement in the portal phase, and multiple vascular shadows penetrating, and patchy non-apparently strengthened low-density shadow area, and some of the generative branches of intrahepatic vein and portal vein were not clearly shown, with the change of the left branch of the middle hepatic vein and hepatic vein (**Figure 1D**), suggesting that it was a neoplastic lesion. It was a tumor lesion, not excluding hepatoblastoma. After completing the relevant examinations, an ultrasound-guided ultrasonic tumor aspiration biopsy of the liver was performed under general anesthesia, and postoperative symptomatic supportive treatment was provided. The postoperative puncture pathology result was considered as hepatic infantile hemangioma (HIH). Due to the combination of severe hypothyroidism and the huge tumor, which occupied almost the entire liver, and the overall poor health condition, in October 2023, the child underwent allogeneic living liver transplantation.

**Figure 2:**
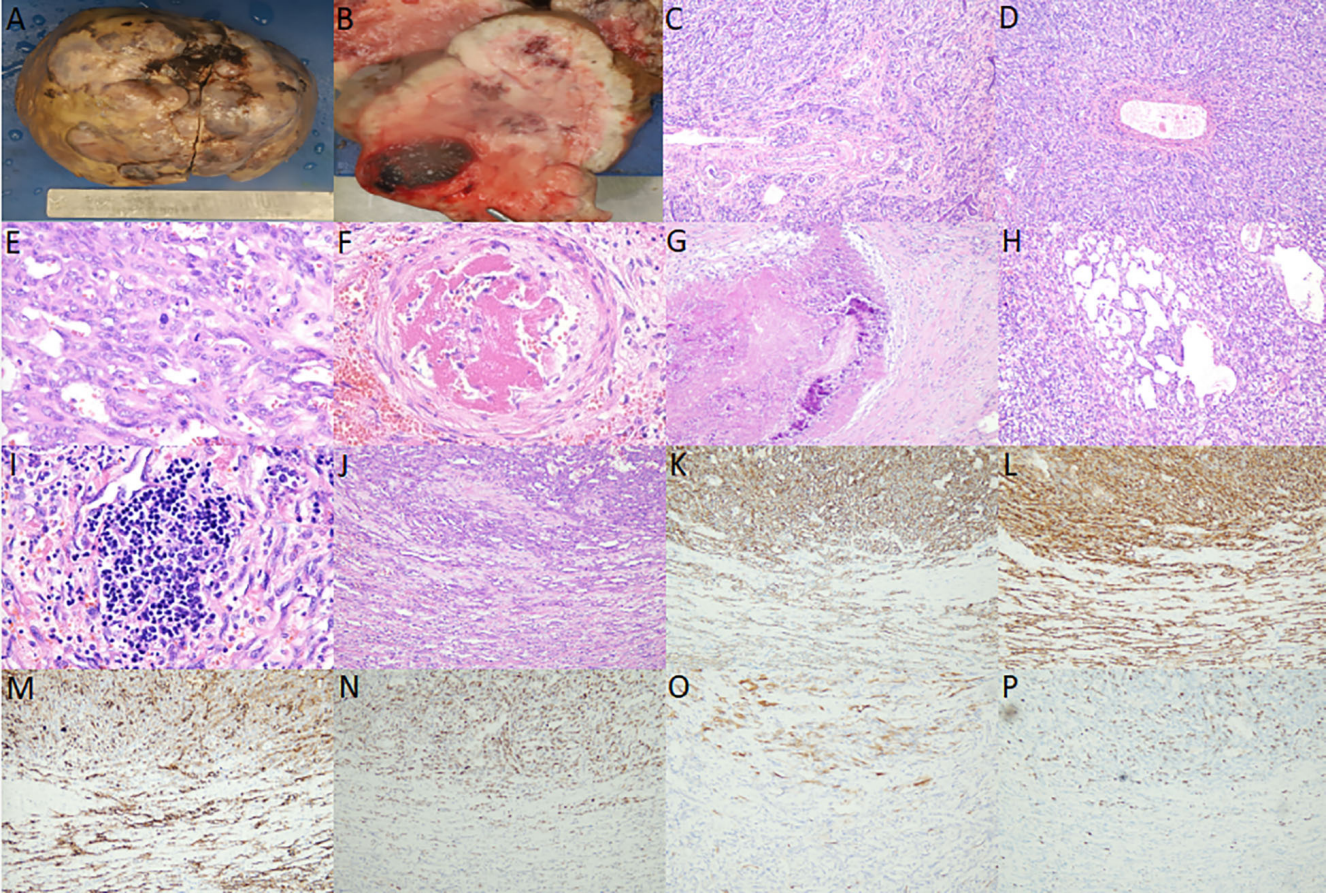
**(A)** Gross view shows that the liver is enlarged in size, dysmorphic, and partially elevated on the surface; **(B)** Section view shows that the mass is grayish white, grayish yellow, hard, and hemorrhage and necrosis are observed; **(C, D)** hepatic infantile hemangioma (HIH) microscopy shows that the tumor cells are arranged in the form of stripes, tubulointerstitial or fissure, and the tumor shows extensive irregular bile ducts with reactive proliferation, etc. (medium magnification); **(E)** Hepatic infantile hemangioma (HAS) microscopy shows that the vascular lumens have anastomosed with each other to form a sieve mesh, and the tumor is characterized by heterogeneous proliferation of cells, enlarged nuclei and pathologic nuclear schizophrenia (high magnification); **(F)** intra-arterial thrombus (high magnification); **(G)** Necrosis, fibrosis, and calcification (medium magnification); **(H)** Cavernous angiomatous foci (medium magnification); **(I)** Extramedullary hemopoiesis (high magnification); **(J)** Microscopically, HIH and HAS lesions are continuous, without any clear boundaries, and the upper part of the lesions is the HAS region, and the lower part is the HIH region (medium magnification); **(K, L)** Cluster of differentiation (CD) 31 and CD34 positivity in the upper half of the HAS region and the lower half of the HIH region (medium magnification); **(M)** Glucose transporter protein isoform 1 positivity in the upper half of the HAS region and the lower half of the HIH region (medium magnification); **(N)** Diffuse and strong positivity for P53 in the upper half of the HAS region, and individual weak positivity for P53 in the lower half of the HIH region (medium magnification); **(O)** P16 positivity in the upper half of the HAS region and P16 negativity in the lower half of the HIH region (medium-fold); **(P)** Increased Ki-67 proliferative activity in the upper half of the HAS region (approximately 20% positivity rate) and lower Ki-67 proliferative activity in the lower half of the HIH region (approximately <1% positivity rate) (medium-fold).

Liver transplant specimen had a total size of 18.0 × 16.0 × 11.5 cm, weighing 1699.6 g. The liver was enlarged in size, dysmorphic, with a partially elevated surface, and a mass was observed on the cut surface, measuring approximately 16.5 × 14.0 × 7.0 cm, with a greyish-white and greyish-yellow hard cut surface. Hemorrhage and necrosis were observed, and the demarcation from the surrounding hepatic tissue was clear, without obvious envelope (**Figures 2A, B**).

There was no obvious fibrous envelope around the tumor. Low magnification showed that the tumor cells were arranged in the form of stripes, tubular lumen or fissure, infiltrating and destroying normal liver tissues, and the remnants of the confluent area and central vein could be observed, and the tumor showed extensive irregular bile ducts, reactive hyperplasia. High magnification showed that the tumor cells were flattened to the short spindle, with inconspicuous anisotropy, and delicate/peppery chromatin, without obvious nucleoli. Pathological nuclear schizophrenia was rare, and diffuse and scattered neutrophils were also observed. Diffusely scattered neutrophils, a few lymphocytes, plasma cells, and eosinophils infiltrated the tumor (**Figures 2C, D**); some areas of the vascular lumens anastomosed with each other to form a sieve mesh, and pseudopapillae and solid areas were observed. The tumor cells were heterogeneous and proliferative, with spindle-/or epithelioid-like morphology, uneven chromatin distribution, enlarged nuclei, and pathologic nuclear schizophrenia, and hemangiosarcoma was formed (**Figure 2E**). Thrombosis was observed in the vasculature of individual arterioles (**Figure 2F**). Necrosis, fibrosis, and calcification were visible in the periphery (**Figure 2G**), and areas of cavernous hemangiomas could be observed at multiple places (**Figure 2H**) along with extramedullary hematopoiesis (**Figure 2I**). HIH and hepatic angiosarcoma (HAS) were continuous microscopically, with no obvious boundaries (**Figure 2J**).

HIH and HAS regions were positive for cluster of differentiation (CD) 31, CD34, and glucose transporter protein isoform 1 (GLUT-1); HIH regions were individually weakly positive for P53, negative for P16, and had low proliferative activity of Ki-67 (positivity rate of approximately <1%), whereas HAS regions were diffusely strongly positive for P53, positive for P16, and had increased proliferative activity of Ki-67 (positivity rate of approximately 20%) (**Figures 2K-P**). HHV-8, D2-40, GS, Desmin, S100, SALL4, CD3, and CD20 were negative. HepPar-1 and Arg-1 showed residual hepatocytes, and CK8/18, CK19 showed hyperplastic bile ducts.

The child had severe hypothyroidism as a comorbidity. The tumor was large, and the patient’s overall physical condition was poor. Therefore, the decision was made for the patient to undergo a liver transplantation. After the child underwent an allogeneic living donor liver transplantation, he was routinely administered oral tacrolimus for anti-transplant rejection with blood concentration monitoring. The patient exhibited hypothyroidism both before and after liver transplantation and was consistently managed with oral levothyroxine sodium tablets. The patient was routinely followed up with coagulation, liver function, renal function, blood glucose, and blood lipids testing, and ultrasound by liver transplantation specialists. Until April 14, 2025, after more than 18 months of postoperative follow-up, there was no recurrence or metastasis, and the patient’s general condition remained good. Thyroid function progressively normalized by the fifth postoperative month.

## 3 Discussion

A retrospective analysis of previously reported cases involving malignant transformation of HIH into HAS revealed that the initial diagnosis age ranged from 4 months to 7 years, with an average age of approximately 34.7 months. The male-to-female ratio was 13:5. 88.8% of patients presented with an abdominal mass, while the remaining cases presented with abdominal pain, jaundice, or fever. One case was associated with a skin hemangioma, and one case had a history of arsenic exposure. Treatment modalities included radiotherapy, chemotherapy, hepatic artery embolization, and surgical resection. One case did not receive treatment. The survival period ranged from 0 to 4 years, with an average survival period of approximately 20.7 months. Patients who underwent surgical treatment had a longer survival period than those who did not undergo surgical treatment.

On imaging, it is easily misdiagnosed as other tumors, such as hepatoblastoma and mesenchymal mesothelioma. Color ultrasound showed that the mass was a solid hypoechoic area, and a liquid hypoechoic area could be observed in the center. CT scan showed round, round-like, or irregular low-density foci, and it could be high density when accompanied by hemorrhage and calcification. The edges of the mass in the arterial phase of the enhancement scan showed the “double-ring sign.” MRI showed that the tumor had a more homogeneous signal, and it could be caused by hemorrhage, necrosis, and fibrosis if it was combined with hemorrhage. When combined with hemorrhage, necrosis, and fibrosis, the signal is uneven (11). Dynamic enhancement MRI has been shown to be highly specific for HIH diagnosis owing to its characteristic enhancement pattern (12), and the early “erythema” of Tc-99m RBC liver scintigraphy is also a specific diagnostic feature for HIH (13). Imaging has no risk of bleeding and can be repeated. Some studies have reported that the diagnostic accuracy of ultrasound for HIH can reach 98.1%, whereas the diagnostic accuracy of CT and MRI can reach 96.2%; therefore, the preferred diagnostic method for HIH is still imaging. The performance of ultrasound and CT examination of the child in this case was consistent with previous reports. However, due to the rarity of the lesion and the lack of awareness of HIH, it was misdiagnosed as hepatoblastoma, and a puncture biopsy was incorrectly performed. Furthermore, malignant transformation into HAS occurred in this case, which reminds imaging physicians that, while accurately suggesting HIH, attention should be paid to changes in imaging findings that are suggestive of malignant transformation.

HIH appears as a whitish-brown nodule in the gross view. It can be categorized into three types according to the extent of liver parenchyma affected: focal, multifocal, or diffuse lesions (14). Focal HIH is usually only detected on prenatal imaging and is not considered a true HIH. Multifocal HIH presents with areas of hemangioma intervening in the normal hepatic parenchyma. Diffuse HIH is defined as a tumor that almost completely replaces the hepatic parenchyma. In our case, the child had a diffuse lesion, which showed a huge solid occupancy in almost the whole liver, and hemorrhage and necrosis were visible in the section, which was clearly demarcated from the surrounding liver tissues without obvious coating.

Histomorphologically, HIH comprises malformed and dense capillaries, the lumen of which is coated with a thin layer of proliferating endothelial cells, with mucus stroma and bile ducts, with rare nuclear schizophrenia, and large spongy hemangiomatous foci in the center of larger lesions, with intravascular thrombosis and necrosis, secondary fibrosis and calcification, and extramedullary hematopoiesis in some cases. Among them, fibrosis, calcification and formation of spongy angiomatous foci are representative of tumor degeneration or maturation. When malignant transformation into HAS occurs, the vascular lumens anastomose with each other to form a sieve mesh. Pseudo-papillae and solid areas are observed, with heterogeneous hyperplasia of tumor cells, spindle/or epithelioid morphology, uneven distribution of chromatin, enlarged nuclei, and pathologic karyorrhexis are easily observed. In this case, the main area of the liver was HIH, and part of the area was HAS, and the two lesions were continuous without obvious boundaries, which proved that the HAS area was a malignant transformation of the HIH area. Intravascular thrombus, necrosis, fibrosis, calcification, foci of cavernous hemangioma and extramedullary hematopoiesis were also observed in this case, consistent with previous reports.

GLUT-1 is a HIH-specific marker, which is expressed in precursor endothelial cells, and postnatal expression sites are limited to the blood-tissue barrier. It is also observed in erythrocytes and neural fasciculocytes, which can be used to differentiate between HIH and other hemangiomas and vascular malformations. GLUT-1 is expressed in HIH and is absent in other tumors and vascular malformations; however, it cannot be used as a diagnostic marker for benign and malignant HIH and HAS. In this case, both HIH and HAS regions were positive for GLUT-1, suggesting that HIH was the underlying lesion. The HAS region was diffusely strongly positive for P53, strongly positive for P16, and showed significantly elevated proliferative activity of Ki-67 (positivity rate of approximately 20%), whereas the opposite was true for the HIS region. This suggested that the HAS region was malignantly transformed from the HIH region, and the immunophenotype was consistent with previous reports.

To summarize the above features, HIH and the malignant transformation of HIH into HAS have little clinical and imaging differences, and it is extremely difficult to distinguish them based on imaging. The malignant transformation of HIH into HAS can be diagnosed when the following pathological conditions are met: contains both HIH and HAS regions, both lesions are continuous under the microscope, the vascular lumen of the HAS region has anastomosed with each other to form a sieve mesh, pseudo-papillae and solid zones can be observed, the tumor cells are heterogeneously proliferated with spindle/or epithelioid morphology, the chromatin distribution is uneven, the nuclei are enlarged, and the pathologic karyorrhexis is easily visible. GLUT-1 has specific characteristics in HIH and HAS, and it is very difficult to distinguish between them by imaging. P53, P16, and Ki-67 were highly expressed in the HAS region, whereas the opposite was true for the HIS region.

HIH and its malignant transformation into HAS need to be differentiated from other common tumors of the infant’s liver. (1) Hepatoblastoma (HB): It is the most common hepatic malignant tumor in children. Nearly 90% of HB cases occur within the age of 5 years. Microscopy shows that the tumor comprises epithelial and mesenchymal metaplasia components at different stages of maturation, and vasculogenic markers are negative. (2) Mesenchymal hamartoma (MH): It is the second most common benign tumor in children, with 85% of the cases occurring within the age of 2 years. Its incidence is slightly higher in male children than in female children. Its occurrence may be related to abnormal development of the bile duct plate and mesoderm during embryonic development. Its clinical manifestations are similar to those of HIH, and imaging shows intrahepatic multicompartmental cystic with solid septations, largely grayish yellow, with cystic degeneration. The cystic lumen is filled with yellow fluid or jelly-like material. Microscopically, it comprises different proportions of loose connective tissues and choledocholithiasis or choledocholithiasis-like components, and choledocholithiasis is often twisted or dilated, and the interstitial stroma is commonly edematous, and mucinous degeneration may occur (15). The dilated choledochal ducts are CK7-positive, and the areas of cystic degeneration are negative for vasculogenic markers and CK7. (3) Congenital hepatic hemangioma: It is less common than HIH and is currently considered to be a congenital vascular malformation with capillary proliferation. It proliferates *in utero*; the tumor is formed before birth and does not increase in size after birth. It can rapidly regress on its own, and in the process of regression, a large area of central necrosis and calcification can occur. Imaging suggests arteriovenous malformation (16). Negative expression of GLUT-1 has diagnostic significance. (4) Metastatic tumor of the liver: This is most commonly observed to occur due to neuroblastoma metastasis. Diffuse or multiple nodules are observed in the liver. Microscopically, it is a small round cell tumor, and a characteristic daisy-shaped cluster structure can be observed. Immunohistochemical markers are positive for Syn, CgA, and S100, whereas vasculogenic markers are negative.

At present, there is no uniform treatment guideline for this type of tumor in HIH, and a reasonable individualized treatment plan should be formulated according to the specific situation of each patient. The treatment plan mainly includes conservative observation, drug therapy, surgical resection, and liver transplantation. For asymptomatic children with HIH, conservative treatment involves observation under color ultrasound monitoring, waiting for its natural regression (17). For multiple tumors or uncomplicated cases, drug treatment can be administered, including steroid hormones, interferon therapy, propranolol, chemotherapeutic drugs, etc. As propranolol was used in the treatment of hemangiomas in 2008, some scholars recommend propranolol as a first-line drug for the clinical treatment of HIH (18). China also released the Expert Consensus on the Use of Oral Propranolol Treatment of Infantile Hemangiomas in 2016 and updated it 2022. According to this consensus, symptomatic patients are given symptomatic treatment along with medication, and surgical resection is feasible when necessary, with a better prognosis. Liver transplantation is feasible if the tumor is huge and serious complications exist. When malignant transformation of HIH into HAS occurs, there is a lack of consensus on whether treatment for HIH should be extended.

In conclusion, although HIH can spontaneously regress, it is necessary to prevent potentially life-threatening complications, and complex multimodal treatment is required (19, 20). There is a lack of case reports related to the malignant transformation of HIH into HAS. Further, it is not clear whether the malignant transformation can be detected by imaging, whether the malignant transformation into HAS can only be diagnosed pathologically, and whether HIH treatment should still be continued, and the prognosis is also unclear. More case reports are needed to accumulate experience.

## Data Availability

The raw data supporting the conclusions of this article will be made available by the authors, without undue reservation.
